# Transcriptome sequencing based annotation and homologous evidence based scaffolding of *Anguilla japonica* draft genome

**DOI:** 10.1186/s12864-015-2306-6

**Published:** 2016-01-11

**Authors:** Yu-Chen Liu, Sheng-Da Hsu, Chih-Hung Chou, Wei-Yun Huang, Yu-Hung Chen, Chia-Yu Liu, Guan-Jay Lyu, Shao-Zhen Huang, Sergey Aganezov, Max A. Alekseyev, Chung-Der Hsiao, Hsien-Da Huang

**Affiliations:** Institute of Bioinformatics and Systems Biology, National Chiao Tung University, Hsin-Chu, 300 Taiwan, ROC; Computational Biology Institute & Department of Mathematics, George Washington University, Washington, DC, USA; Department of Higher Mathematics, ITMO University, St. Petersburg, Russia; Department of Bioscience Technology, Chung Yuan Christian University, Chung-Li, Taiwan, ROC; Department of Biological Science and Technology, National Chiao Tung University, Hsin-Chu, 300 Taiwan, ROC; Department of Biomedical Science and Environmental Biology, Kaohsiung Medical University, Kaohsiung, Taiwan

**Keywords:** *Anguilla japonica*, Scaffolding, RNA-seq, Transcriptome, Genome annotation

## Abstract

**Background:**

*Anguilla japonica* (Japanese eel) is currently one of the most important research subjects in eastern Asia aquaculture. Enigmatic life cycle of the organism makes study of artificial reproduction extremely limited. Henceforth genomic and transcriptomic resources of eels are urgently needed to help solving the problems surrounding this organism across multiple fields. We hereby provide a reconstructed transcriptome from deep sequencing of juvenile (glass eels) whole body samples. The provided expressed sequence tags were used to annotate the currently available draft genome sequence. Homologous information derived from the annotation result was applied to improve the group of scaffolds into available linkage groups.

**Results:**

With the transcriptome sequence data combined with publicly available expressed sequence tags evidences, 18,121 genes were structurally and functionally annotated on the draft genome. Among them, 3,921 genes were located in the 19 linkage groups. 137 scaffolds covering 13 million bases were grouped into the linkage groups in additional to the original partial linkage groups, increasing the linkage group coverage from 13 to 14 %.

**Conclusions:**

This annotation provide information of the coding regions of the genes supported by transcriptome based evidence. The derived homologous evidences pave the way for phylogenetic analysis of important genetic traits and the improvement of the genome assembly.

**Electronic supplementary material:**

The online version of this article (doi:10.1186/s12864-015-2306-6) contains supplementary material, which is available to authorized users.

## Background

Abundance of Japanese eel, as well as other freshwater eels belongs to genus *Anguilla*, has been radically shrinking in the past decade [1]. Catadromous eels’ enigmatic life cycle makes their reproduction affected deeply by anthropogenic impacts. Lack of an economical method to artificial reproduce makes this organism extremely vulnerable to overconsumption. Mature eels migrate thousands of kilometers into the open ocean to spawn eggs. Exact spawning locations of Japanese eels were hard to pinpoint and remained unknown until recently when they were found near Western Mariana Ridge. What prohibits the research from further improvement is that, eggs and larvae of *Anguilla japonica* are spread by the Kuroshio Current, making the habitats spans a wide area of Eastern Asia [2]. Cylindrical shape larva develops into transparent color leptocephalus larvae, which eventually metamorphosis into glass eels. Glass eels migrate back into the freshwater through estuaries, sometimes traveling within wet sands into the inner continent, where they spend years going through pigmentation into yellow eels, and then silver eels [3]. Such wide area of habitation potentially makes effect of pollution and diseases to be accumulated. Long life cycle and the spawning habit through migration make wild Anguilla eels hard to recover from the damage caused by overfishing.

Physical linkage map of Japanese eel were constructed in 2011 [4]. High throughput Sequencing technology was rarely applied upon this issue before. However, with the advances of sequencing technologies bringing down the cost and time consuming of DNA and RNA sequencing, plus the approaching extinction of fresh water eels, the field began to change. In 2010, mRNA-Seq study of deep sequencing and de novo reconstruction of European glass eel were reported as well as the hox genes of the specie [5], 2 years following that, draft genome sequence of European were also published [3]. The incorporative research of genomic and transcriptomics information from the deep sequencing should have major impacts in multiple fields. Expression profiling of both transcriptome of European eels response to environmental pollution were reported in 2012 [6]. The first draft genome of Japanese eel was assembled [7], proving that the hox genes and genomic distance of European and Japanese eels were conserved. By 2014, a ddRAD-based linkage map was published, providing 13 % coverage of the draft genome [8]. Such results left plenty of space for improvement.

Hereby, we provide a reconstructed transcriptome from whole body samples of juvenile (glass eels). The high throughput sequencing provides unprecedented amount of transcriptomic information. Instead of focusing only on certain types of tissues or organs, full transcriptome of the entire organism was sequenced. For future experimental design and guidance on ecological, physiological, artificially breeding and even toxicity resistance study of Japanese eel, such transcriptome can provide additional guidance. What‘s more, the massive amount of evidence provided by the transcriptome helps the complete of draft genome structural annotation. Combining transcriptome sequence data with publicly available expressed sequence tags evidences, 18,121 genes were structurally and functionally annotated on the draft genome. The structural annotation was performed through an established pipeline, MAKER [9]. Functional annotation was based on sequence alignment. The acquired homologous evidences were further used to improve the draft genome scaffolding. Applying an improved version of scaffolding algorithm developed by Aganezov et al. [10], synteny of *Anguilla japonica* was compared to the genome of Fugu, Stickleback, Medaka, Tetraodon, Coelacanth and Zebra fish. Obtained results were integrated with previously published linkage map [8], putting 3,921 genes into the 19 linkage groups, which represent chromosomes of *Anguilla japonica*. 137 scaffolds were grouped into the linkage groups in addition to the original partial linkage groups. Phylogenetic analysis of the gene clusters correlation to thyroid hormone receptors and pigmentation were performed with MEGA 6.0 [11].

## Results and discussion

Sequencing through Illumina HiSeq^TM^ 2000 generated total 85,233,812 reads, with length of 101 nucleotides. After quality control, low quality reads were trimmed and left 77,939,562 reads were left with an average length of 99.575 nucleotides (Additional file 1: Figure S1). Quality control of the sequence reads is summarized in Additional file 1: Figure S2 and Additional file 1: Table S3. Assembly were assessed through average length of unigenes, as well as quality score N50 and N90. As the result shows in Additional file 1: Table S2, average length, N50 and N90 of clustered unigenes are significantly higher than results of single De Novo assembly tools. Composition of assembled unigenes showed in Additional file 1: Table S3 demonstrates that clustered unigenes tends to have higher composition of longer nucleotides. Hence assembles generated through clustering were considered to have higher accuracy and were used for further annotation. In Additional file 1: Figure S2, we demonstrate that expression level measured with FPKM, frequency of reads per kilo base per million, distributes through all different length of assembles.

Summary of the functional annotation is listed in Additional file 1: Table S4. From the total 32,210 assembled unigenes, 16,106 were found to be aligned to known proteins in NCBI Non Redundant protein database. 10,848 of the unigenes were found to contain functional domains through RPSBLAST against NCBI Conserved Domain Database. 5641 transcripts were found to involve with system biological pathway in Kyoto Encyclopedia of Genes and Genomes (KEGG) [12]. 13,434 transcripts were annotated to certain Gene Ontology terms. Up to top 5 blast hits per query were considered in the process. Distribution of homologs belonging to other organisms were illustrated in Additional file 1: Figure S3.

Expression level of the transcripts was examined together with their frequency to be assigned to certain Gene Ontology terms. As demonstrated in Additional file 1: Figure S4, transcripts regarding enzyme regulator activity express level exceeded the total average expression level despite the fact that only four of them were found. Distribution of assembled transcripts through different KEGG pathways categories (Additional file 1: Figures S5 and S6) were also observed alongside with average expression level. As shows in Additional file 1: Figure S5, despite only few transcripts found in some pathway categories such as Circulatory system and Reaction module maps, average expression level of transcripts within these categories demonstrates potential rich activities of these pathways.

Protein functional domains found on the assembled transcripts were also viewed in their distribution alongside with expression level, demonstrated in Additional file 1: Figures S7 and S8. Protein functional domains with extreme expression level can provide guidance to the future protein- protein interaction study.

Catadromous eels’ reproduction is limited by their long life cycle and migration spawning. To produce enough supply without consuming wild glass eels, development of technology that would shorten the period of time for eels to mature, and would artificially induced spawning of healthy larvae is inevitable. Hence, revealing the mechanisms of metamorphosis from leptocephalus larvae into glass eels, as well as fermentation from glass eels to mature silver eels is the key to successful artificial reproduction to supply commercial demands and keep wild eels from extinction.

In the past, transcriptomic studies of eels mainly relied on classical molecular biological experimental methods. Studies of various mechanisms were performed with classical molecular methods such as cloning and protein purification. Cloning and protein purification provide only partial view of the transcripts [1]. To fully capture all protein coding transcripts, a combination of next generation sequencing and new transcript assembly algorisms is necessary [13]. In 2014, a study of mRNA expression profile through RT-PCR of prolactin, growth hormone, and somatolactin of Japanese eel was reported [14]. However, researches utilizing genome information of Japanese eel, and the respective resources available for the experimental design are still limited. On the other hand, hybrids of European and American eels were found occurred naturally in Iceland [15], import of European glass eels into East Asia could trigger interspecific hybridization of *Anguilla* eel, inducing further anthropogenic impacts to this species near extinction [1]. Proven possibilities of hybrid reproduction [15], as well as the successful artificial hybrid of European and Japanese eels also bring new possibilities to the artificially reproduction [16]. However, the transcriptomic information is still limited. In 2013, the first transcriptomic study through 454 deep sequencing was performed on gill of *Anguilla japonica* [17]. Utilization of proteomic approaches and transcriptomic sequencing gave insights into the osmoregulation mechanism, providing transcriptomic view of Anguilla japonica’s catadromous behavior. However the study [17] didn’t correlate with the currently available draft genome.

On eel sexualize mechanism, several surveys have been conducted on ovarian steroid genesis [18]. Expression level of several genes were also found to be related to the ovarian development. Through the attempts of artificial reproduction of glass eels has been attempted since 1930s in Europe [1], only until 2003, first successful artificially induced spawning of Japanese eel was achieved through injection of salmon pituitary extracts into the female eel and human chorionic gonadotropin into male eel [19]. An unpublished successful F2 generation was declaimed in 2011 [4]. However current technology is not sufficient for large scale reproduction. Mortality of artificially cultured eels is still high.

Under current circumstance, all main stream studies of Japanese eels should inevitably focus on how to successfully improve life cycle of eels under the artificial environment to suit the existing demand. Such studies would take into consideration with all kinds of mechanisms. Metamorphosis, pigmentation and sexualize mechanisms of eels are all deeply correlated to their catadromous spawning activities, especially the metamorphosis mechanisms including reorganization of the entire body plan. Complete genome structure and transcriptome is essential for future study.

To suite such a purpose, we provide the first complete transcriptome of glass eels. Application of deep sequencing provides not only the information of homologs, but also the potential novel genes of Japanese eels. Clustering of De Novo assembled transcripts from different tools through overlapping successfully increase the assembly quality. Distribution of assembled transcripts through different species, GO terms, system biological pathway and protein functional domains of found genes were examined and demonstrated. In addition, we further provide expression level alongside the distributions. Such demonstration successfully provides the hidden information about pathways with few genes but extreme expression levels.

**Fig. 1:**
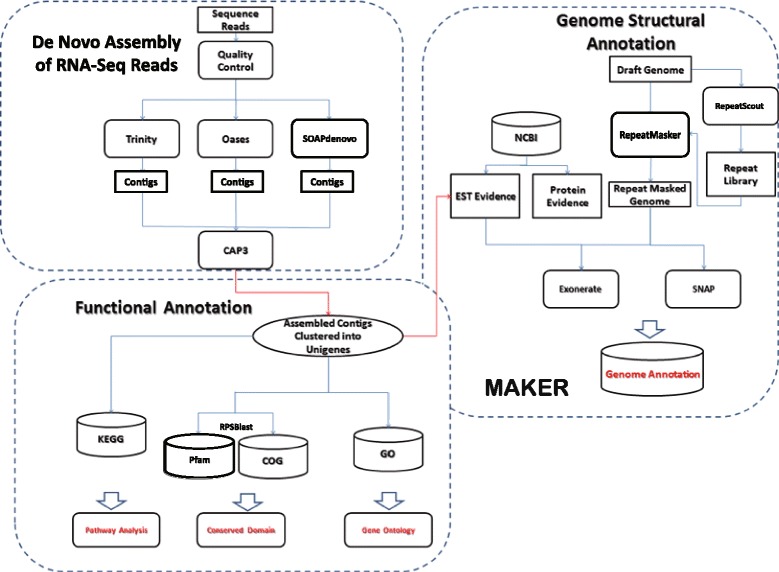
Overview of the Genome Annotation Process. The overall flowchart of the genome annotation of *Anguilla Japonica* is showed in this graph. De Novo assembly of the transcriptome was treated as EST evidences, combined with other previously published information, aligned onto the draft genome. The annotation process was performed through MAKER

Without structural and functional annotation, draft genome [7] provides only limited information since *Anguilla japonica* is not an established model organism [20]. The massive amount of information provided by RNA Sequencing for our experiment makes transcriptome evidence sufficient enough to perform complete structure annotation, which identifies genes and their intron-exon structure. Before doing so, due to the fact that Eukaryotic genomes are rich with repeat sequences, a process of repeat masking needs to be carried out. Usually such a process would need a well-established repeat sequence library to serve as guideline. However such a library doesn’t exist for *Anguilla japonica*. In this case [20], de novo constructing a new library [21] from the draft genome is a better choice than using established model organism data base such as Repbase [22]. Structural annotation supported by RNA-seq could be done by directly assembling reads on to the genome with tool like Cufflinks. We further improved the de novo assembly into a genome based assembly following the convention guideline [20]. An overview of the annotation process can be found in Fig. 1. The automatic pipeline we used, MAKER [9], performs multiple ab initio gene predictions, and cross verifies with the evidence driven prediction. Such prediction, backed up by the rich evidences, increase the accuracy of the structural annotation.

**Fig. 2:**
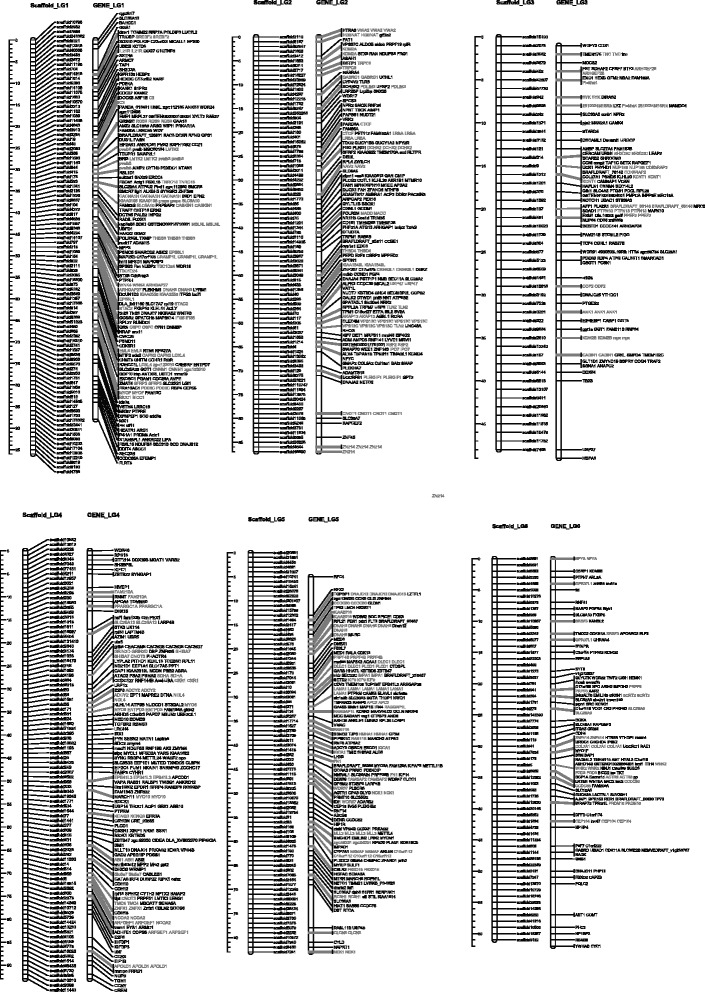
Homolog Linkage Map of *Anguilla Japonica* (Linkage Groups 1 to 6). The graph shows genetic linkage maps on 6 of the 19 linkage groups. With the homologs synteny information derived from the functional annotation, we successfully allocated 137 scaffolds (13 Mb) into the established linkage group. This graph illustrates the relative position of the scaffolds. Order of the combined scaffolds was determined by an application of topological sort to combine the linkage maps of male and female linkage. Since evidence of the distance between scaffolds is not available, only the putative order was demonstrated here. The homologs gene cluster was showed in gray color

**Fig. 3:**
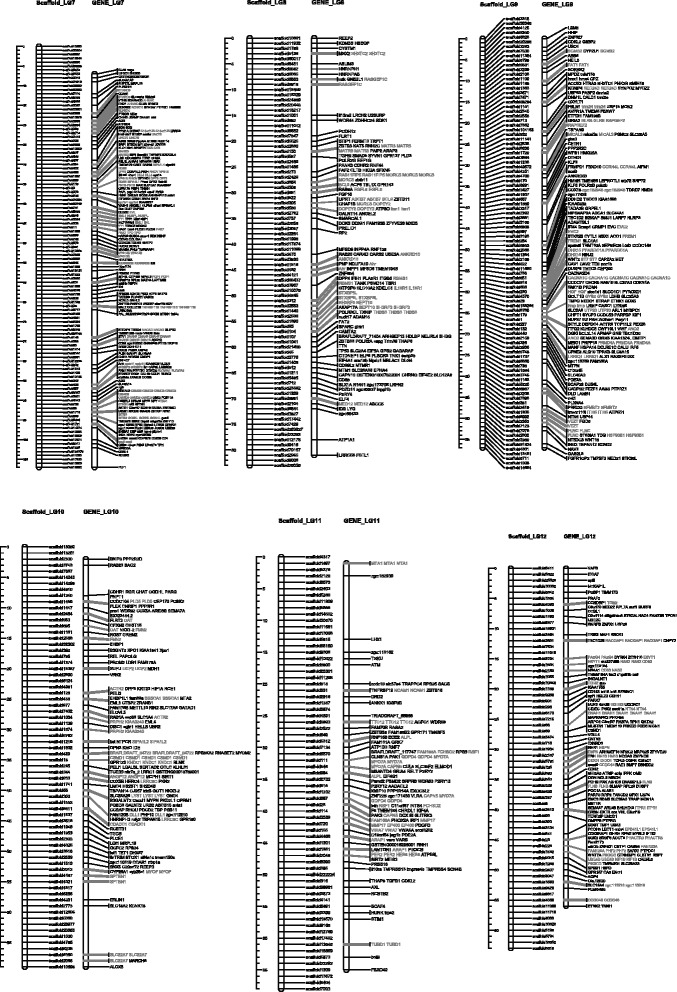
Homolog Linkage Map of *Anguilla Japonica* (Linkage Groups 7 to 12). The graph shows genetic linkage maps on 6 of the 19 linkage groups. With the homologs synteny information derived from the functional annotation, we successfully allocated 137 scaffolds (13 Mb) into the established linkage group. This graph illustrates the relative position of the scaffolds. Order of the combined scaffolds was determined by an application of topological sort to combine the linkage maps of male and female linkage. Since evidence of the distance between scaffolds is not available, only the putative order was demonstrated here. The homologs gene cluster was showed in gray color

**Fig. 4:**
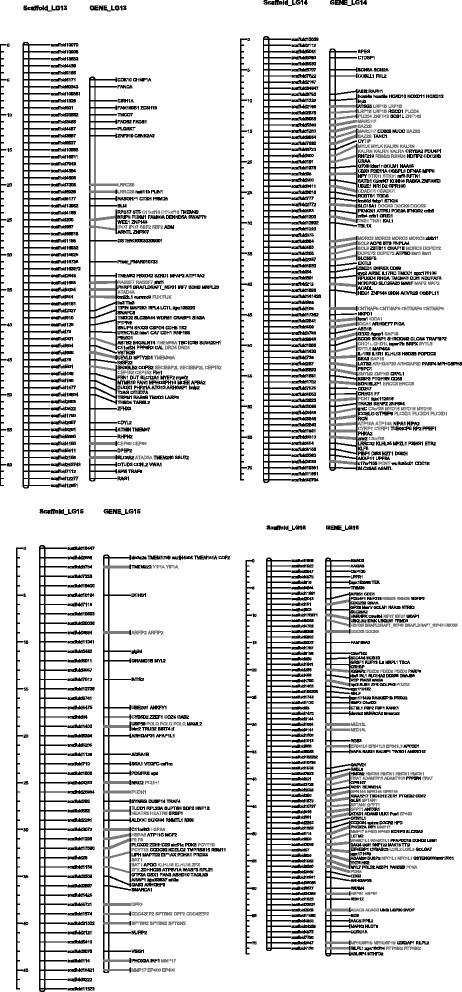
Homolog Linkage Map of * Anguilla Japonica* (Linkage Groups 13 to 16). The graph shows genetic linkage maps on 4 of the 19 linkage groups. With the homologs synteny information derived from the functional annotation, we successfully allocated 137 scaffolds (13 Mb) into the established linkage group. This graph illustrates the relative position of the scaffolds. Order of the combined scaffolds was determined by an application of topological sort to combine the linkage maps of male and female linkage. Since evidence of the distance between scaffolds is not available, only the putative order was demonstrated here. The homologs gene cluster was showed in gray color

**Fig. 5:**
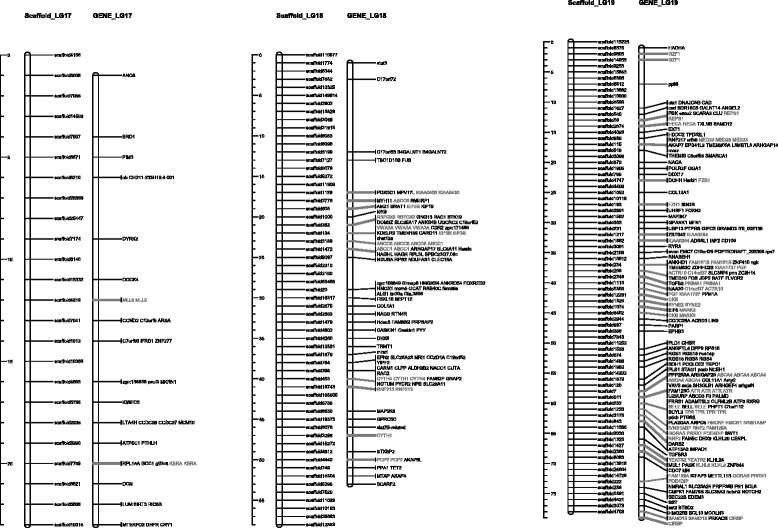
Homolog Linkage Map of *Anguilla Japonica* (Linkage Groups 17 to 19). The graph shows genetic linkage maps on 3 of the 19 linkage groups. With the homologs synteny information derived from the functional annotation, we successfully allocated 137 scaffolds (13 Mb) into the established linkage group. This graph illustrates the relative position of the scaffolds. Order of the combined scaffolds was determined by an application of topological sort to combine the linkage maps of male and female linkage. Since evidence of the distance between scaffolds is not available, only the putative order was demonstrated here. The homologs gene cluster was showed in gray color

Many assemblies of the bone fish draft genomes were built upon SNP base linkage groups. With the homologs synteny information derived from the functional annotation, we successfully allocated 137 scaffolds (13 Mb) into the established linkage group. The relative position of the homologs and clusters on these linkage map are illustrated in Figs. 2, 3, 4 and 5. The plots were generated with Mapchart [23].

**Fig. 6:**
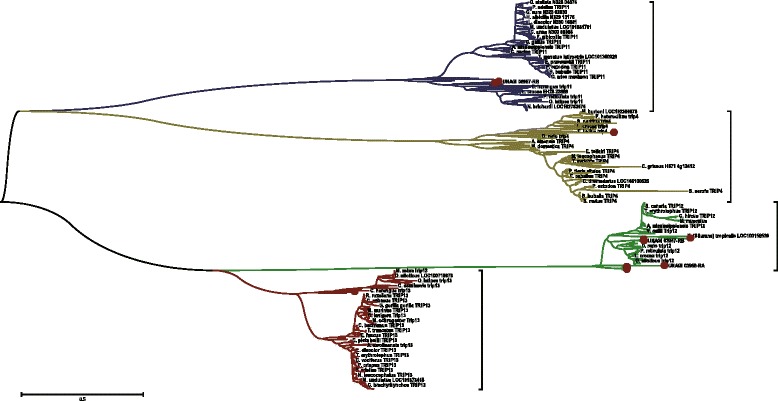
Phylogenetic Tree of Thyroid Hormone Receptor Interactor (TRIP) Family Homologs. This graph illustrated the thyroid hormone receptors homologs of Anguilla Japonica. From the separate color subtree of TRIP4, TRIP11, TRIP12 and TRIP13 genes homologs, we can see that the different homologs of same gene family are distributed into different subtree

**Fig. 7:**
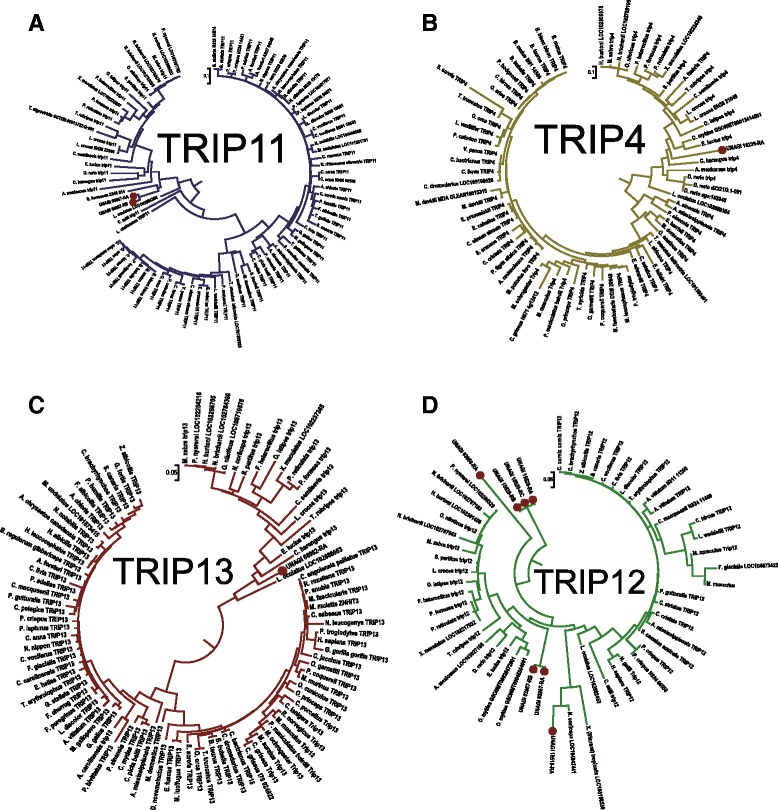
Phylogenetic Trees of Thyroid Hormone Receptor Interactor (TRIP) Subfamilies. In this graph, each circular tree represents the corresponding color subtrees illustrated in Fig. 6. **a** The blue circular tree represent the TRIP11 subtree, with 2 *Anguilla Japonica* homologs marked in red dots. **b** The yellow circular tree represent the TRIP4 subtree, with 1 Anguilla Japonica homologs marked in red dot. **c** The red circular tree represent the TRIP13 subtree, with 1 Anguilla Japonica homologs marked in red dot. **d** The green circular tree represent the TRIP12 subtree, with 7 Anguilla Japonica homologs marked in red dots**.** Through the analysis we found that the thyroid hormone receptor interactor family genes of Japanese eel are homologous to Asian arowana, Northern Pike, Rainbow trout, Marbled rockcod, Atlantic herring and Spotted gar

**Fig. 8:**
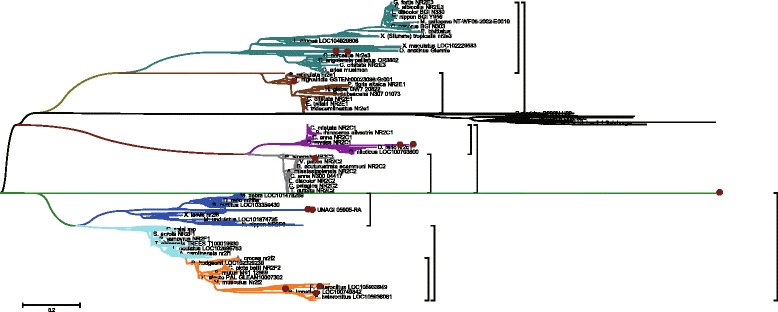
Phylogenetic Tree of Nuclear Receptor Subfamily 2. This graph illustrated the nuclear receptors homologs of *Anguilla Japonica*. From the separate color circular subtree of NR2F2, NR2F6, NR2C1, NR2C2, NR2E1 and NR2E3 genes homologs, we can see that the different homologs of same gene family are distributed into different subtree

**Fig. 9:**
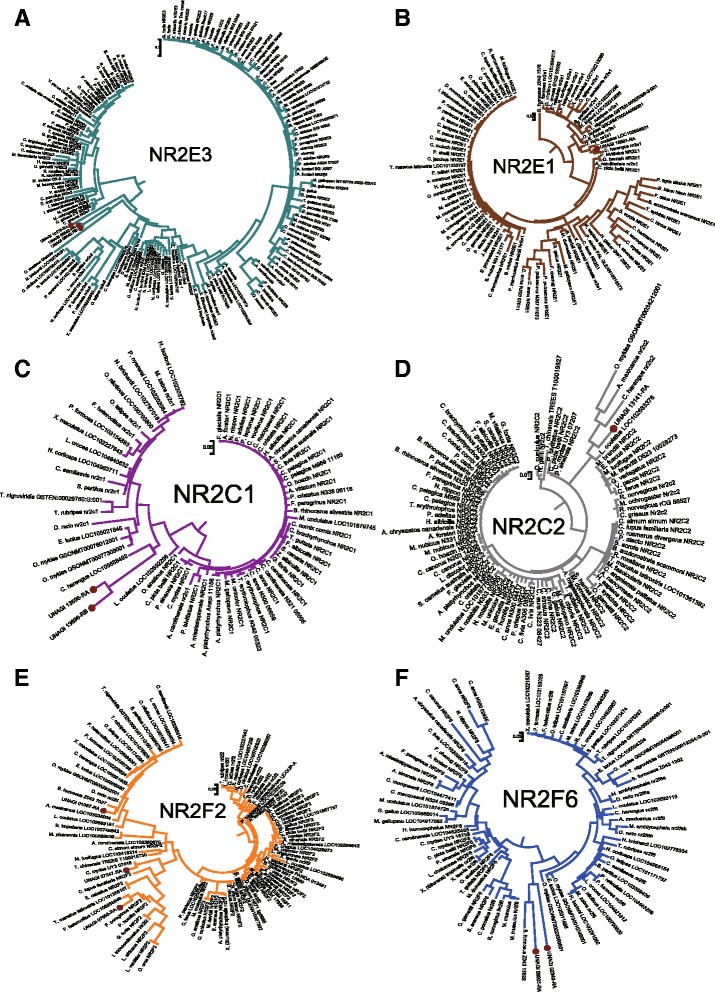
Phylogenetic Trees of Nuclear Receptor Subfamily 2 Groups. In this graph, each circular tree represents the corresponding color subtrees illustrated in Fig. 8. **a** The green circular tree represent the NR2E3 subtree, with 3 *Anguilla Japonica* homologs marked in red dots. **b** The brown circular tree represent the NR2E1 subtree, with 1 *Anguilla Japonica* homolog marked in red dot. **c** The purple circular tree represent the NR2C1 subtree, with 2 *Anguilla Japonica* homologs marked in red dots. **d** The gray circular tree represent the NR2C2 subtree, with 1 *Anguilla Japonica* homolog marked in red dot**. e** The yellow circular tree represent the NR2F2 subtree, with 3 *Anguilla Japonica* homologs marked in red dots**. f** The blue circular tree represent the NR2F6 subtree, with 2 *Anguilla Japonica* homologs marked in red dots**.** Through the analysis we found that the thyroid hormone receptor interactor family genes of Japanese eel are homologous to Asian arowana, Northern Pike, Rainbow trout, Marbled rockcod, Atlantic herring and Spotted gar

We hypothesis that metamorphosis mechanism of Anguilla japonica, like other Teleost fishes, conserve with amphibians [24], based on the fact that development and growth of fish correlates to thyroid hormone had been widely accepted [25]. Such metamorphosis of vertebrate tends to be triggered by environmental control on hypothalamo-pituitary-thyroid axis in the brain [24], and regulated by the thyroid hormone receptor on cell membrane. The environment control, in this case, would be the catadromous activity from ocean to inland. In certain developmental stages of the larval, the brain senses the environmental stress urging the metamorphosis to glass eel, and releases corticotropin release factor (CRF), which correlated with genes crh and CRHBP. CRF binds on receptors of anterior pituitary, forcing it produce thyrotrophin (TSH) and adrenocorticotrophic hormone (ACTH). TSH triggers thyroids to secret thyroid hormone, while ACTH triggers adrenals or Interrenals to secret corticoids. From our functional annotation, we observed that the trha homolog of Japanese eel is correlated with TSH, and pomcb homolog is correlated with ACTH of Anguilla japonica. Finally, thyroid hormone receptors on the cell membrane as well as nuclear receptors within the cell regulate whether the metamorphosis would be triggered. We believe that thyroid hormone receptors of *Anguilla japonica* are regulated by the TRIP4, THRAP3, TRIP11, TRIP12, TRIP13 genes homologs, while the nuclear receptors are regulated by the genes NR4A3, NR2C2, NCOA3 and NR2F6 homologs respectfully. Phylogenetic tree of thyroid hormone receptor interactor (TRIP) family homologs is illustrated in Figs. 6 and 7. Through the analysis we found that the thyroid hormone receptor interactor family genes of Japanese eel are homologous to Asian arowana, Northern Pike, Rainbow trout, Marbled rockcod, Atlantic herring and Spotted gar. Phylogenetic tree of nuclear receptor subfamily 2 is illustrated in Figs. 8 and 9. From the graph we observed that the receptors were also closely related to homologs of same six fishes, Asian arowana, Northern Pike, Rainbow trout, Marbled rockcod, Atlantic herring and Spotted gar.

**Fig. 10:**
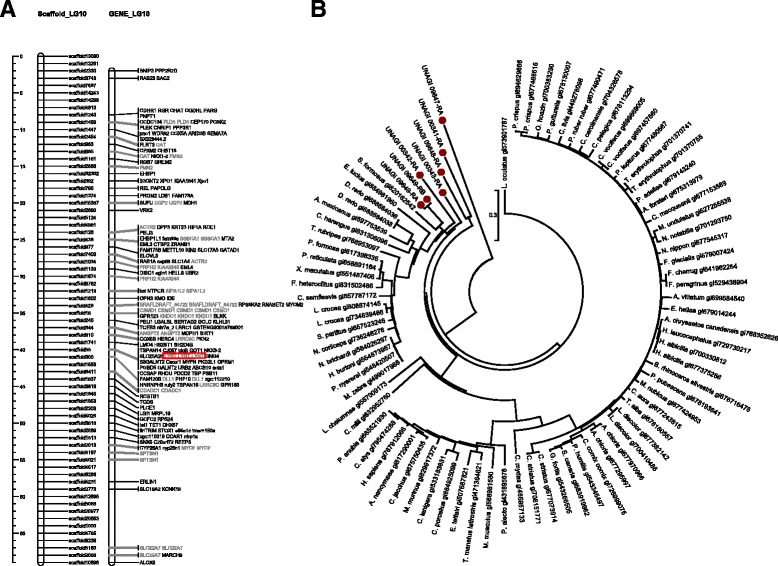
Phylogenetic Tree and Linkage Location of LYST Homologs. In this graph, we found a lysosomal trafficking regulator LYST cluster (in red box) in linkage group 10 (LG10). The gene was close related to the homolog of Asian arowana. There are 4 other Anguilla Japonica homologs of LYST marked on the circular tree

Among the homolog clusters we have found, lysosomal trafficking regulator LYST cluster in linkage group 10 (LG10) was related to the GO term pigmentation GO:0043473 [26]. Correlated scaffolds in the linkage group and phylogenetic analysis of the found homologs are illustrated in Fig. 10. From the phylogenetic tree we found that LYST homolog of Japanese eel is also close related to Asian arowana, a kind of freshwater East Asia fish with high commercial value.

While such a full transcriptome from whole body is proven to be effective on functional annotation, scope of this annotation is still limited by the sample. Since the messenger RNAs were isolated from glass eels before sexualization, certain types of hormones from gender specific tissues of silver eels such as ovarian and testicular cannot be found in such samples. On the other hand, satellite sequences and mRNAs from eggs and larval might not necessary been expressed in our samples. Despite some of a portion of this information is available on NCBI, which we used in our annotation, these factors could still limit the completeness of the annotation.

## Materials and methods

Isolated whole RNA from the five glass eels were prepare for the RNA sequencing. Libraries for the RNA-Seq were sequenced through Illumina HiSeq^TM^ 2000 following the manufacturer’s manual. Pair end libraries were sequenced in 101 X 2 nucleotides length reads, with 120 nucleotides adaptors. The entire fragment length was 357 nucleotides. The base calling and image analysis were done following Illumina standard pipeline. Raw reads of deep sequencing went through quality control procedure done by using FASTX-Toolkit: FASTQ/A short reads pre-processing tools, with only Quality value Phred score over 20 nucleotides remain, which means only reads with per base accuracy over 99 % were kept. Also, we trimmed the length of the reads down to 70 nucleotides for low quality reads. The transcriptome were first reconstructed through de novo assembly. To achieve maximum accuracy, we applied three different main stream de novo assembly tools : Trinity , Oases [27], and SOAPdenovo-Trans [28]. Quality controlled reads were assembled into three separate sets of contigs, with the three different tools. Trinity was applied with default settings, while Oases and SOAPdenovo-Trans were applied with multiple-kmers strategy. To further eliminate overlapping contigs, we clustered the three sets of contigs with CD-HIT-EST [29] into three sets of unigenes. Finally, we clustered unigenes with high similarity together with the tool CAP3 [30]. Quality of the assembly was estimated mainly through an average length of unigenes, as well as quality score N50 and N90. N50 represents the length of the longest unigene among the collection of unigenes equal to a half of the sum of all unigenes, while N90 means the length of the shortest unigene among the collection of unigenes equal to ninety percent of the sum of all unigenes. Maximum and minimum length of assembled unigene also serves as an index for the assessment.

Abundance of the assembled unigenes were estimated through RSEM pipeline [31]. Quantities of the transcripts were estimated through FPKM value. FPKM, frequency of reads per kilo base per million ,value was calculated through aligning reads onto assembled transcripts with Bowtie [32]. A de novo repeat library of *Anguilla japonica* was built from the draft genome [7] through RepeatScout [21]. Then, de novo assembly of the RNA-Seq data were pooled with the complete and partial CDS, EST and previously done gill RNA-seq assembly [17] of *Anguilla japonica* from NCBI as EST evidence. The EST evidence includes the public available RNA-seq data sets SRX482728, SRX247092, SRX115953 and the whole body transcriptome sequence data. Together with the known proteins from NCBI, genome structural annotation was performed through the pipeline MAKER [9]. The pipeline firstly masked the repeat sequences according to the previously build library with Repeatmasker (http://repeatmasker.org), and then perform ab initio prediction through repeat training of SNAP [33] and polished with Exonerate [34].

To find the assembled transcripts coding proteins, unigenes were blasted against NCBI non-redundant protein data base, TrEMBL and Swiss-Port [35] with BLASTX. Hits with an e value lower than 10 to negative 5, filtered by penalty estimation through the credibility of the protein, would be considered as homologs. Next, available Gene Ontology [36] terms were found listed. On the other hand, potential protein conserved domain were found through RPSBLAST against Pfam [37] and NCBI COG . To help the system biological analysis in the future, available KEGG pathways [38] were also annotated.

**Fig. 11:**
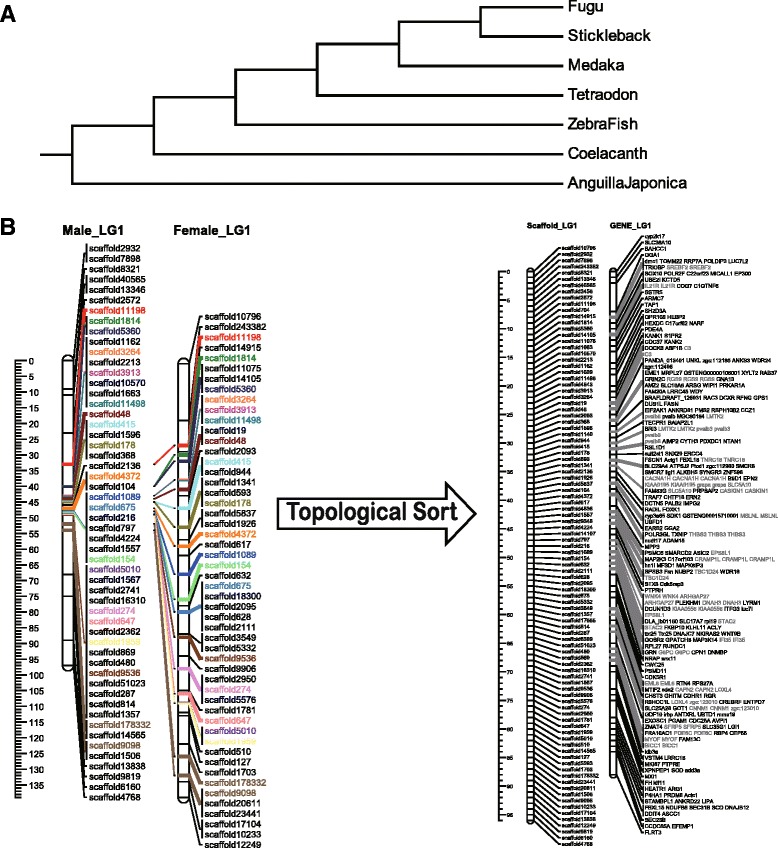
Overview of the Genetic Linkage Map Building Process. **a** We utilized the following phylogenetic tree. While provided with 7190 scaffolds with homologous genes on them, scaffolding method identified 525 links and assembled scaffolds respectively. These scaffolds were then mapped into male and female linkage maps provided by the Kai et al. study. **b** The scaffolds mapped into male and female linkage groups were then sorted into single group. As illustrated in the graph, scaffolds marked with same colors provide the evidences for the general order of them on the chromosome. The order can then be sorted through topological sort algorithm

For scaffolding purposes, we utilized an improved version (to be described elsewhere) of the gene order based scaffolding method developed by Aganezov et al. [10]. Since this method relies on gene orders of multiple genomes, we preprocessed genomic sequences of Fugu [39], Stickleback [40], Medaka [41], Tetraodon [40], Coelacanth [42] and Zebra fish [43] in addition to *Anguilla japonica* to represent them as sequences of homologous gene (decided by respective scaffolds boundaries). Scaffolds with no homologous genes were filtered out from genomes during the preprocessing. In contrast to the original method described in Aganezov et al. [10], the improved method accounts for gene insertions/deletions and duplications and thus no filtration for unique gene content was needed. We utilized the phylogenetic tree in Fig. 11 a. While provided with 7190 scaffolds with homologous genes on them, scaffolding method identified 525 links and assembled scaffolds respectively. These scaffolds were then mapped into male and female linkage maps provided by the Kai et al. study [8]. Order of the combined scaffolds was determined by an application of topological sort. With the scaffolds grouped into male and female linkage groups overlapped with each other, order of the scaffolds on the chromosomes can be sorted with topological sort algorithm. Phylogenetic tree of the fishes and the process of topological sort are illustrated in Fig. 11.

## Conclusions and prospective works

We provide a reconstructed transcriptome from whole body samples of juvenile (glass eels). The high throughput sequencing provides unprecedented amount of transcriptomic information. For future experimental design and guidance on ecological, physiological, artificially breeding and even toxicity resistance study of Japanese eel, the transcriptome provide guidance. For example, expression of specific genes shows extreme patterns in glass eels and can be further compared with larvae as well as silver eels through QPCR to provide further reevaluation of the metamorphosis and pigmentation mechanism.

### Availability

Link to a gtf file of the annotation, fasta files of the protein and transcripts and genetic linkage map would be available in Additional file 1.

## Additional files

Additional file 1:
**Contains a link to the gtf file of the annotation, fasta files of the protein and transcripts and genetic linkage map.** Additional file 1 also includes Figure S1 to Figure S8, Table S1 to Table S4. (DOCX 429 kb)
