# Supplementary material for: Transcriptome sequencing based annotation and homologous evidence based scaffolding of Anguilla japonica draft genome
Source: BMC Genomics. 2016 Jan 11;17(Suppl 1):13. doi: 10.1186/s12864-015-2306-6 (PMC4895481; doi:10.1186/s12864-015-2306-6)
Supplement: Additional file 1: — Contains a link to the gtf file of the annotation, fasta files of the protein and transcripts and genetic linkage map. Additional file 1 also includes Figure S1 to Figure S8, Table S1 to Table S4. (DOCX 429 kb) [file 12864_2015_2306_MOESM1_ESM.docx]

**Additional file 1**

A gtf file of the annotation, fasta files of the protein and transcripts and genetic linkage map is available in:

[**https://www.dropbox.com/sh/in3vtr4utlntjpo/AAAnD369B65hfAM93WUUTp63a?dl=0**](https://www.dropbox.com/sh/in3vtr4utlntjpo/AAAnD369B65hfAM93WUUTp63a?dl=0)


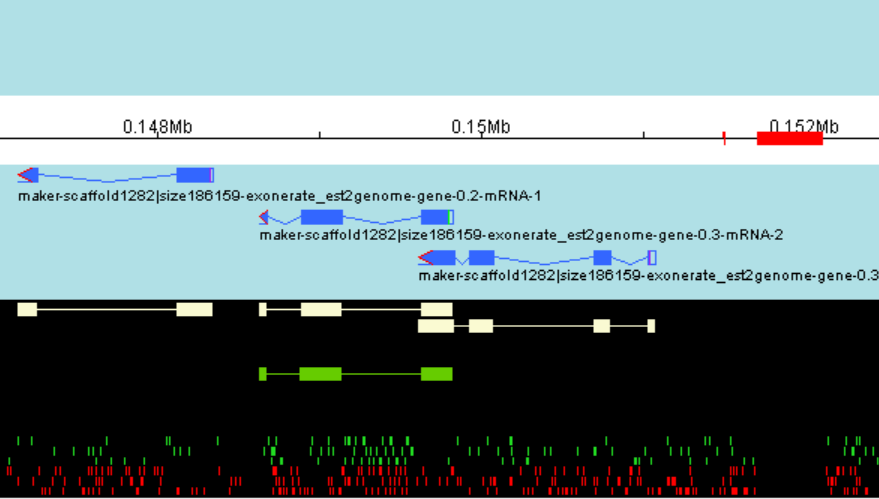


**Figure S1.** Illurstration of Structural Annotation of scaffold1282

**
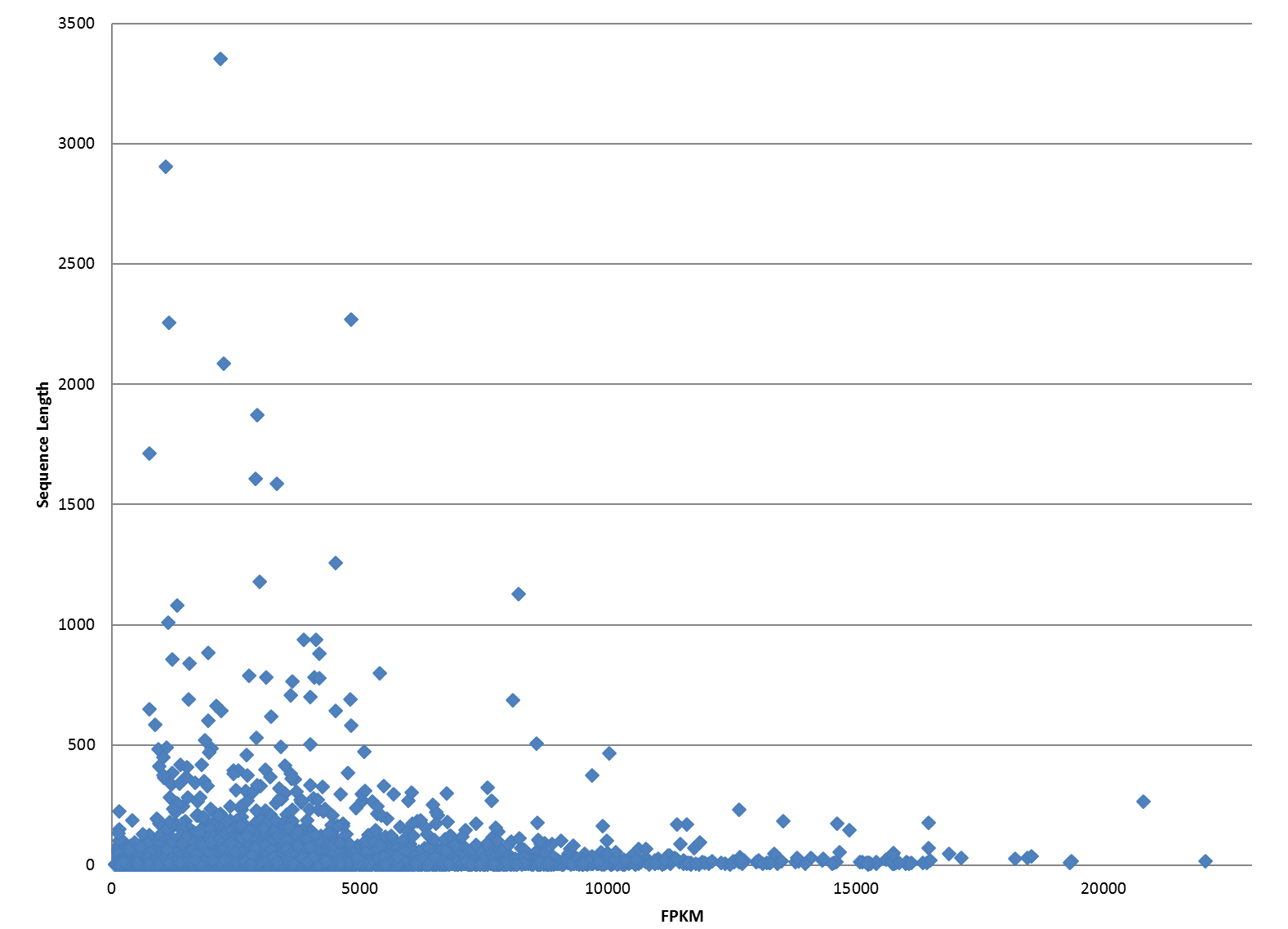
**

**Figure S2.**Relationship between the length and abundance of assembled Unigenes.


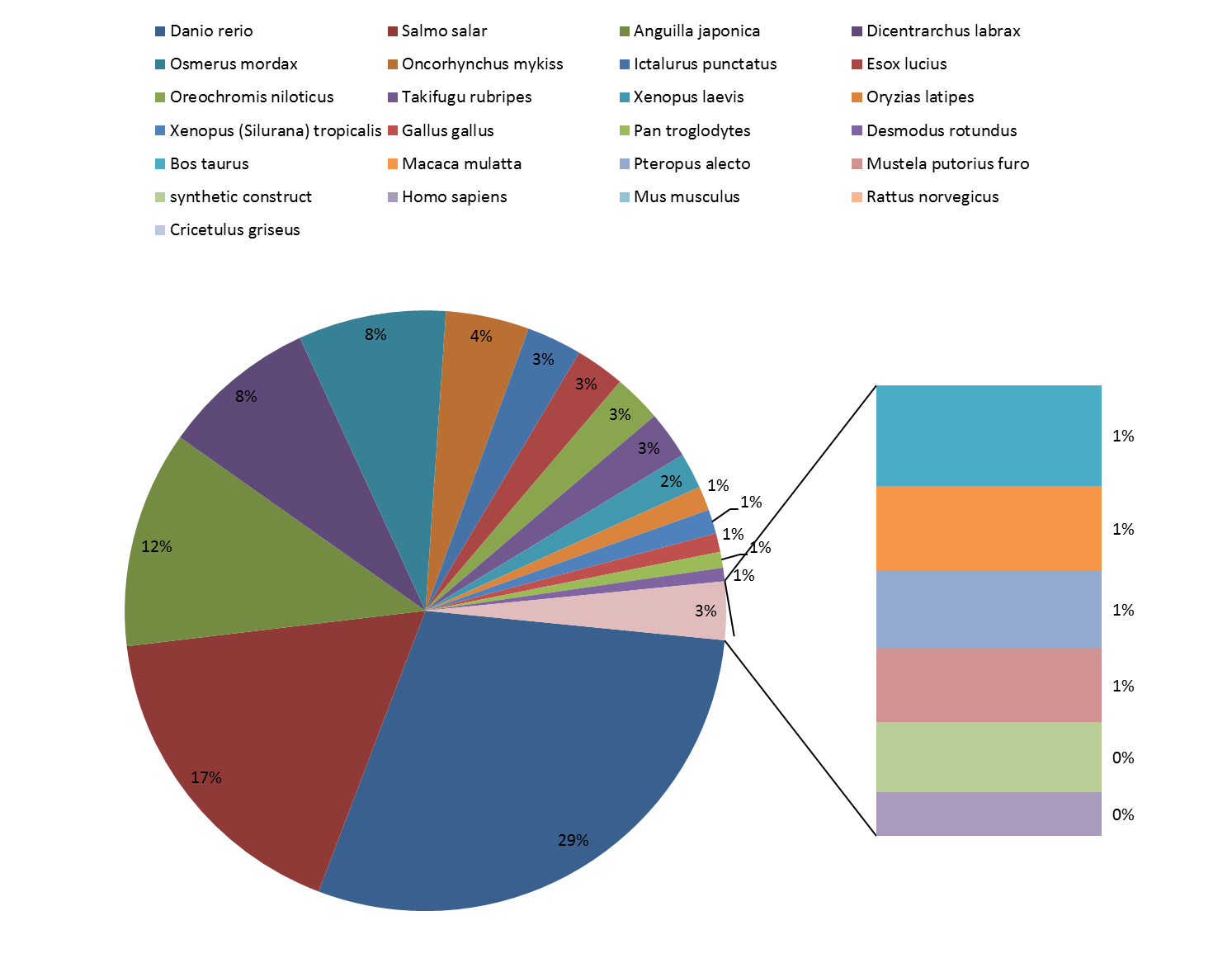


**Figure S3.**Species distribution of top BLASTx results.

**
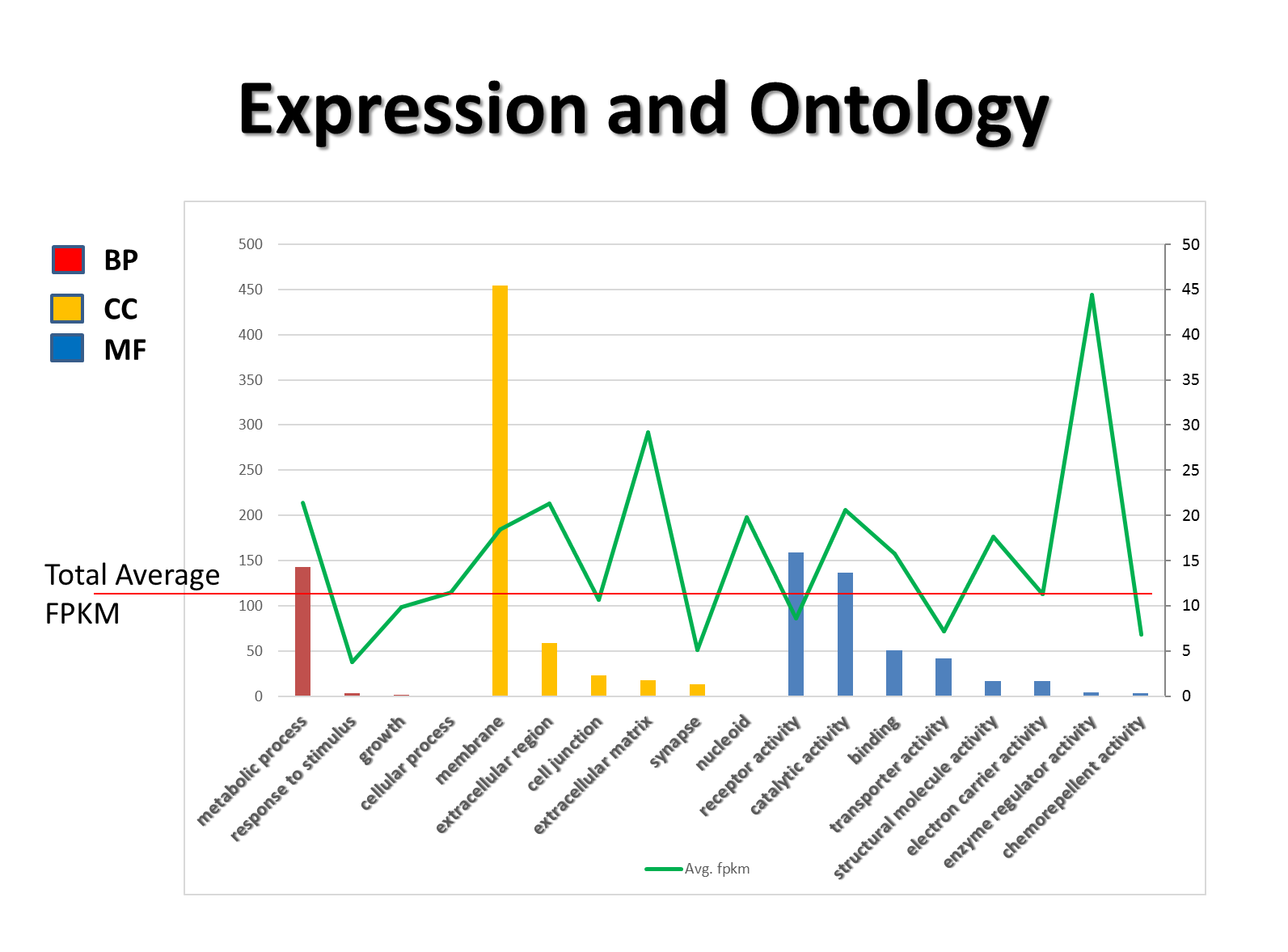
**

**Figure S4.** Gene ontology classifications of assembled Unigenes.

In this figure, amounts of unigenes associated with GO terms were illustrated in the value of y axis. The green line represent the average FPKM of the unigenes associated with the terms illustrated in x axis.

**Figure S5.**KEGG Orthology (KO) classification of assembled unigenes.

In this figure, amounts of unigenes associated with KEGG Orthology terms were illustrated in the value of y axis. The orange line represent the average FPKM of the unigenes associated with the terms illustrated in x axis.

**Figure S6.** Distribution of transcripts on KEGG Orthology (KO) pathways

**Fig S7.** Histogram presentation of clusters of orthologous groups (COG) classification

In this figure, amounts of unigenes associated with clusters of orthologous groups (COG) terms were illustrated in the value of y axis. The orange line represent the average FPKM of the unigenes associated with the terms illustrated in x axis.

**Fig S8.** Histogram presentation of Pfam classification

In this figure, amounts of unigenes associated with Pfam conserve domains were illustrated in the value of y axis. The orange line represent the average FPKM of the unigenes associated with the Pfam family illustrated in x axis.

**TABLES**

**Table S1 Summary of the Illumina HiSeq^TM^ 2000 sequencing result as well as quality control (QC)**

|  | **Read Amount** |
| --- | --- |
| **Total read** | 85,233,812.00 |
| **Read after QC** | 77,939,562 |
|  | Read Length (Nucleotides) |
| **Read Length** | 101 |
| **Average Read Length after QC** | 99.575 |

**Table S2 Length (nucleotides) of the de novo assembled transcripts**

| **Assembly Tool** | **SOAPdenovo** | **Trinity** | **Oases** | **Clustered Unigenes** |
| --- | --- | --- | --- | --- |
| **Total Length** | 167,769,859 | 151,865,571 | 201,936,793 | 60,135,484 |
| **N50** | 150 | 1294 | 2,395 | 3,734 |
| **N90** | 100 | 296 | 510 | 1,024 |
| **Mean** | 150 | 766 | 1,212 | 1,867 |
| **Max** | 3,196 | 16,604 | 22,550 | 22,064 |
| **Min** | 100 | 201 | 100 | 100 |
| **Median** | 105 | 414 | 619 | 931 |

**Table S3 Composition of the assembled Unigenes in length**

| **Assembly Tool** | **SOAPdenovo** | **Trinity** | **Oases** | **Clustered Unigenes** |
| --- | --- | --- | --- | --- |
| **Number of Unigenes<300b** | 93.68% | 32.52% | 28.22% | 25.50%) |
| **Number of Unigenes>=300b** | 6.32% | 67.48% | 71.78% | 74.50% |
| **Number of Unigenes>=1kb** | 0.14% | 21.76% | 37.58% | 49.12% |
| **Number of Unigenes>=2kb** | 0.01% | 8.28% | 19.56% | 36.91% |

**Table S4 Summary of annotation, amount of assembled Unigenes**

| **Annotation** | **Amount of Unigenes** |
| --- | --- |
| **BLASTx against NR** | 16106 |
| **BLASTx against Swissprot** | 15,263 |
| **GO terms** | 13,434 |
| **RPSBLAST against CDD** | 10,848 |
| **COG domains** | 3,594 |
| **Pfam domains** | 9,600 |
| **KEGG ORTHOLOGY** | 5,641 |
